# THE ROLE OF NURSES IN DISORDERS OF CONSCIOUSNESS: ​IMPLEMENTATION OF SENSORY STIMULATION IN THE NEURO-ICU

**DOI:** 10.1093/geroni/igad104.3134

**Published:** 2023-12-21

**Authors:** Rhoodjinie Mentor, Bethany Young, Leah Coghlan, Christopher Boivin, David Fischer

**Affiliations:** University of Pennsylvania, Philadelphia, Pennsylvania, United States; Hospital of the University of Pennsylvania, Philadelphia, Pennsylvania, United States; Hospital of the University of Pennsylvania, Philadelphia, Pennsylvania, United States; Hospital of the University of Pennsylvania, Philadelphia, Pennsylvania, United States; Hospital of the University of Pennsylvania, Philadelphia, Pennsylvania, United States

## Abstract

In the Neuro Intensive Care Unit (Neuro-ICU), patients with Disorders of Consciousness (DoC) often experience sensory deprivation. DoC, resulting from brain damage, encompasses varying degrees of wakefulness and awareness, ranging from coma to vegetative state and minimally conscious state. Research highlights a concerning trend in the Neuro-ICU, where older adults with DoC are more susceptible to mortality compared to their younger counterparts. This study emphasizes the indispensable role of nurses in effectively managing DoC within the Neuro-ICU, particularly through the thoughtful application of sensory stimulation interventions. Sensory stimulation is a promising approach that engages patients’ various senses—touch, sight, sound, and smell, aiming to awaken awareness and cognitive responses in DoC patients. By fostering neural activity, sensory stimulation holds the potential to contribute to their recovery. Importantly, these techniques can be customized to each patient’s specific condition and responses, and nurses, due to their continuous patient interactions, are uniquely positioned to administer these interventions at the bedside. Recognizing this vital role, evidence-based strategies are proposed, encompassing thorough literature analysis, assessment of nurses’ attitudes, and exploration of potential interventions within the Neuro-ICU setting. This research highlights the significant impact nursing can have on elevating patient outcomes. It demonstrates how evidence-based interventions like sensory stimulation can be seamlessly woven into patient care, providing a valuable and holistic approach for individuals grappling with the intricate challenges presented by DoC.

